# 2395. An Interim Report of the Safety, Reactogenicity, and Immunogenicity of a Self-amplifying mRNA (samRNA) COVID-19 Vaccine GRT-R910 as a Booster in Healthy Adults

**DOI:** 10.1093/ofid/ofad500.2015

**Published:** 2023-11-27

**Authors:** Jennifer Whitaker, Paulina Rebolledo, Nadine Rouphael, Getahun Abate, Tara M Babu, Anna Wald, Hana El Sahly, Pedro Garbes, Karin Jooss, Andrew Allen, Lisa McQuarrie, Ranjan Sitaula, Paul C Roberts, Mamodikoe Makhene, Christine M Posavad, M Juliana McElrath, Stephen De Rosa, Rhea Coler, David Montefiori, Amanda Eaton, David M Koelle, Daniel F Hoft

**Affiliations:** Baylor College of Medicine, Houston, Texas; Emory University School of Medicine, Emory University Rollins School of Public Health, Atlanta, GA; Emory University School of Medicine, Atlanta, Georgia; Saint Louis University, St. Louis, MO; University of Washington, Seattle, Washington; University of Washington, Seattle, Washington; Gritstone bio, Inc., Weston, Massachusetts; Gritstone bio, Inc., Weston, Massachusetts; Gritstone bio, Inc., Weston, Massachusetts; The Emmes Company, LLC, Rockville, Maryland; Emmes Company, LLC, Rockville, Maryland; NIH, Rockville, Maryland; NIAID, NIH, Rockville, Maryland; Fred Hutchinson Cancer Center, Seattle, Washington; Fred Hutchinson Cancer Center, Seattle, Washington; Fred Hutch, Seattle, Washington; Seattle Children’s Research Institute, Center for Global Infectious Disease Research, Seattle, Washington; Department of Surgery and Duke Human Vaccine Institute, Durham, North Carolina; Duke University Medical Center, Durham, North Carolina; University of Washington, Seattle, Washington; Saint Louis University, St. Louis, MO

## Abstract

**Background:**

GRT-R910 (Gritstone bio, Inc), a self-amplifying mRNA (samRNA) vaccine expressing the spike protein plus T-cell epitopes of SARS-CoV-2 (D614G variant), was tested in a phase 1 study as a booster in healthy adults.

**Methods:**

This phase 1 open-label, dose escalation study enrolled healthy adults who previously received an approved mRNA COVID-19 vaccine series. Groups of 10 adults aged 18-60 years were boosted with GRT-R910 at 3 or 6 mcg. Adults > 60 years were boosted with GRT-R910 at 3, 6, or 10 mcg. All participants > 60 years in the 6 and 10 mcg dose groups received prior mRNA COVID-19 boosters; none in other groups had prior boosts. Study boosts occurred at least 112 days after completion of primary series/boost of authorized mRNA COVID-19 vaccine or prior SARS-CoV-2 infection. Solicited local and systemic reactogenicity events were collected for 7 days, unsolicited adverse events for 28 days, and serious adverse events (SAEs) for 366 days post-vaccination. Humoral (ELISA IgG against SARS-CoV-2 RBD and neutralizing antibody against D614G and Omicron BA.4/5) are being assessed at multiple time points over 1 year after study vaccination. Participants who self-reported SARS-CohV-2 infection or receipt of non-study COVID-19 booster during the study were censored in the immunogenicity analyses.

**Results:**

No severe local reactogenicity events were observed. Overall, out of 48 enrolled across all groups, 8 reported at least 1 severe systemic reactogenicity event (figure 1). Most severe systemic reactogenicity events were transient, with most graded severe for 1 day or less. No SAEs have been reported. Neutralizing antibody responses remain durable up to 1 year after 3 and 6 mcg boosts in adults 18-60 years (figure 2) and up to 6 months after 3, 6, and 10 mcg boosts in adults > 60 years (data only available through 6 months; figure 3).

**Figure 1:**
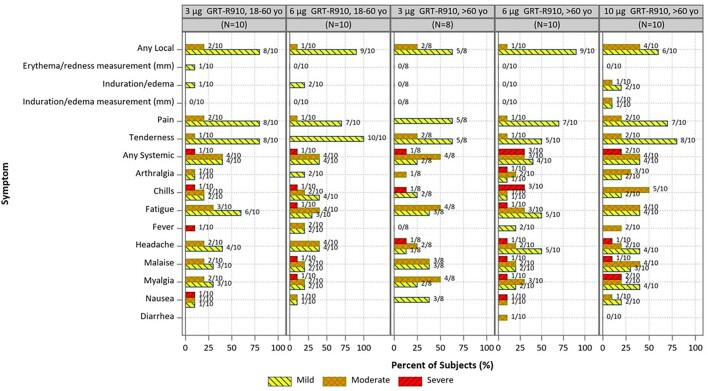
Solicited Systemic and Local Reactogenicity within 7 Days after Receipt of GRT-R910

**Figure:**
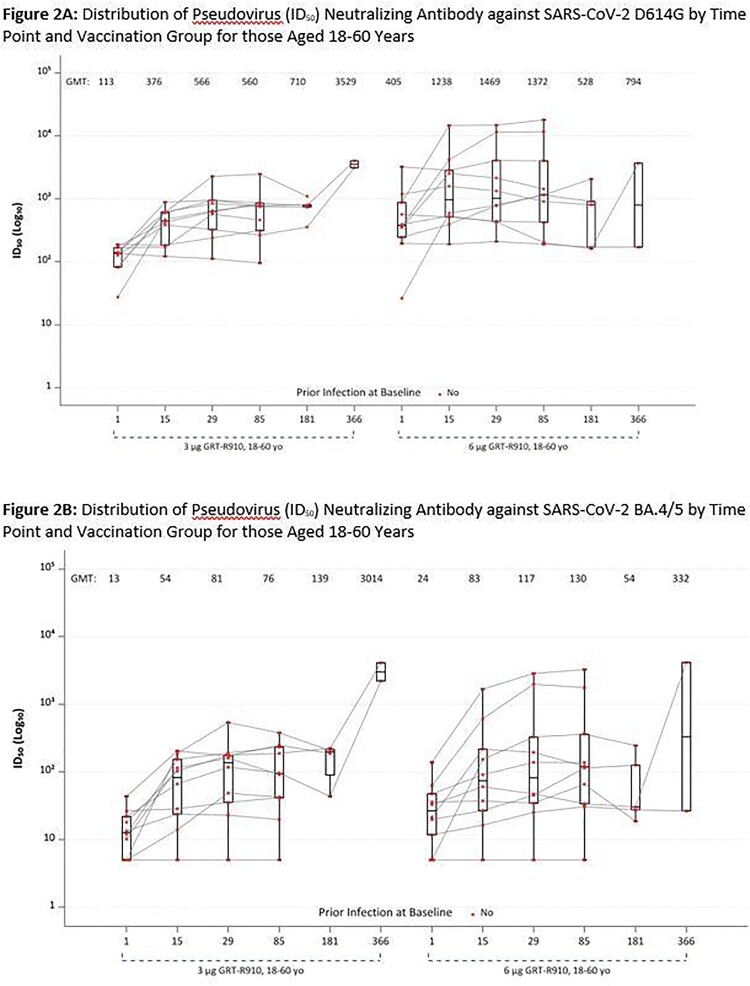

GMT= geometric mean titer; Boxes and horizontal bars denote interquartile range (IQR) and median AUC, respectively. Whisker endpoints are equal to the maximum and minimum values below or above the median +/- 1.5 x IQR. All participants in the 3 μg GRT-R910, 18-60 yo and 6 μg GRT-R910, 18-60 yo groups received a mRNA two dose primary COVID-19 vaccination series prior to enrollment. None were previously SARS-CoV-2 infected.

**Figure:**
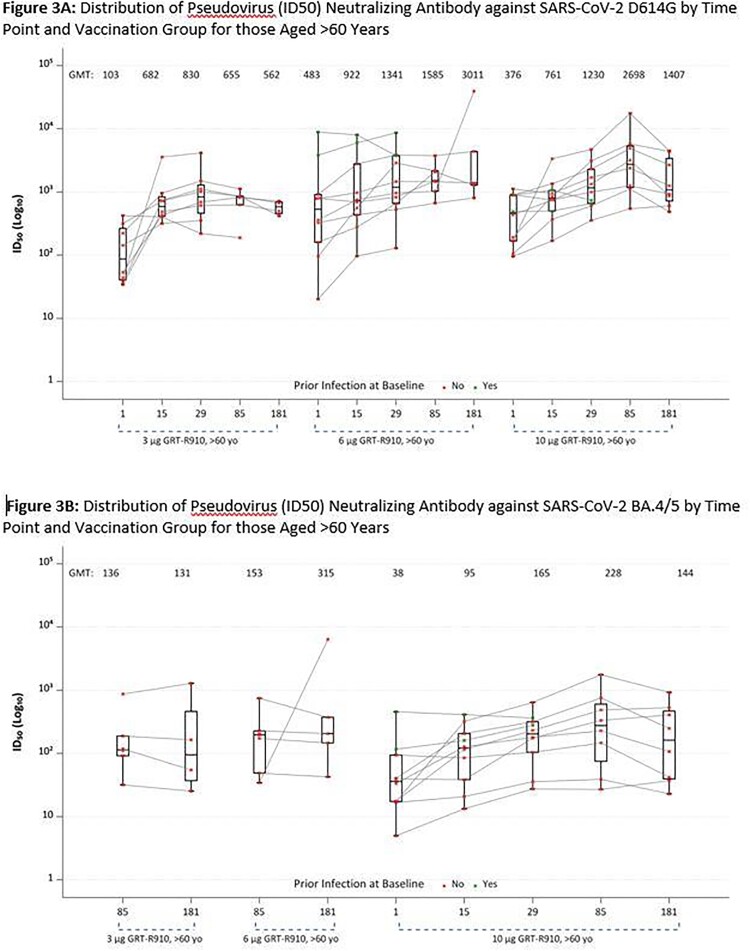

GMT= geometric mean titer; Boxes and horizontal bars denote interquartile range (IQR) and median AUC, respectively. Whisker endpoints are equal to the maximum and minimum values below or above the median +/- 1.5 x IQR. All participants in the 3 μg GRT-R910, > 60 yo group received a mRNA two dose primary COVID-19 vaccination series prior to enrollment. All participants in the 6 μg GRT-R910, > 60 yo and 10 μg GRT-R910, > 60 yo groups received a mRNA two dose primary COVID-19 vaccination series plus a mRNA booster vaccine prior to enrollment. Two participants each in the 6 μg GRT-R910, > 60 yo and 10 μg GRT-R910, > 60 yo groups had a previous COVID-19 infection at enrollment (green dots). D1, D15, D29 testing for Groups 9 and 10 are planned against BA.4/5.

**Conclusion:**

While transient systemic reactogenicity with GRT-R910 as a booster was observed, no safety signals were identified. Preliminary immunogenicity data demonstrate durable neutralizing antibody responses for 6-12 months in both younger and older age groups. Forthcoming T cell response data will aid in assessing the immunogenicity of this novel vaccine.

**Disclosures:**

**Nadine Rouphael, MD**, Icon, EMMES, Sanofi, Seqirus, Moderna: Advisor/Consultant **Anna Wald, MD, MPH**, Aicuris: Advisor/Consultant|Bayer: Advisor/Consultant|Curevo: Participation on Data Safety Monitoring Board|GSK: Grant/Research Support|Sanofi: Grant/Research Support|UpToDate: Royalties or licenses **Pedro Garbes, MD**, Gritstone bio, Inc.: Employee|Gritstone bio, Inc.: Employee|Gritstone bio, Inc.: Stocks/Bonds|Gritstone bio, Inc.: Stocks/Bonds **Karin Jooss, PhD**, Gritstone bio: employee|Gritstone bio: Stocks/Bonds **Andrew Allen, MD, PhD**, Gritstone bio: Board Member|Gritstone bio: Ownership Interest|Gritstone bio: Stocks/Bonds **Amanda Eaton, MBA**, Moderna: Grant/Research Support **David M. Koelle, MD**, Curevo Vaccines: Board Member|MaxHealth LLC: Board Member|Sanofi Pasteur: Grant/Research Support **Daniel F. Hoft, MD, PhD**, Moderna: Advisor/Consultant|Poolbeg: Advisor/Consultant

